# Mechanical and Thermal Properties of Polylactide (PLA) Composites Modified with Mg, Fe, and Polyethylene (PE) Additives

**DOI:** 10.3390/polym12122939

**Published:** 2020-12-09

**Authors:** Zbigniew Oksiuta, Marek Jalbrzykowski, Joanna Mystkowska, Eliza Romanczuk, Tomasz Osiecki

**Affiliations:** 1 Faculty of Mechanical Engineering, Bialystok University of Technology, Wiejska 45C, 15-351 Bialystok, Poland; m.jalbrzykowski@pb.edu.pl (M.J.); j.mystkowska@pb.edu.pl (J.M.); e.romanczuk@pb.edu.pl (E.R.); 2 Institute of Lightweight Structures and Polymer Technology, Chemnitz University of Technology, Reichenhainer Str. 31-33, 09126 Chemnitz, Germany; tomasz.osiecki@mb.tu-chemnitz.de

**Keywords:** hydrolytic degradation, polylactide (PLA) composite, mechanical properties, crystallinity, activation energy of thermal decomposition

## Abstract

In this article, polylactic acid-based composites reinforced with 5% of polyethylene, iron, and magnesium powders were prepared by extrusion and compressed under the pressure of about 10 MPa and characterized. These composites were mechanically, thermally, and morphologically evaluated. It was found, compared to the pure polylactic acid (PLA), an improvement in tensile strength (both σ and YS_0.2_) was obtained for the composite with the iron powder addition, while the magnesium powder slightly improved the ductility of the composite material (from 2.0 to 2.5%). Degradation studies of these composites in the 0.9% saline solution over a period of 180 days revealed changes in the pH of the solution from acidic to alkaline, in all samples. The most varied mass loss was observed in the case of the PLA-5%Mg sample, where initially the sample mass increased (first 30 days) then decreased, and after 120 days, the mass increased again. In the context of degradation phenomenon of the tested materials, it turns out that the most stable is the PLA composite with the Fe addition (PLA-5%Fe), with highest tensile strength and hardness.

## 1. Introduction

Biodegradable and lightweight thermoplastic polymers based on polylactide (PLA) or poly(lactic acid) are becoming more and more popular over the last few years. This is due to the fact that this group of polymers undergo a degradation process, as a result of which they are break down into components that are transformed in the metabolic pathways, without harming the surrounding environment [1,2]. An increase in temperature accelerates the degradation process and at an elevated temperature of about 160–180 °C, polylactide decomposes by 95% in two hours [3].

Composite materials based on the biodegradable polymers (e.g., PLA matrix), due to their unique features and the possibility of structure modification are becoming important materials in the packaging industry, agriculture, horticulture, household, and medical appliances. In the case of polymeric composite materials, the type of reinforcement is a very important issue, since it should not only improve the mechanical properties, but also it should be characterized by biocompatibility and controlled resorbability in a fluid environment.

PLA is a promising thermoplastic aliphatic polyester with relatively high mechanical strength (flexural strength up to 140 MPa, Young’s modulus 5–10 GPa), with excellent optical properties, good processing ability (with low shrinkage not causing product deformation) and complete biodegradation [4,5,6,7,8]. For this reason, these materials have found a number of applications, including the biomedical engineering [9,10]. PLA has also disadvantages, it is a brittle material, and its total elongation in the tensile is about 3%, Charpy impact fracture ~2.5 kJ/m^2^, and it undergo gradual crystallization and enzymatic hydrolysis that limits its application [10]. One of the main requirements for PLA-based composites is a controlled degradation rate, which allows to achieve time-dependent load transfer during its operation and ductility improvement. It is well known that PLA has a biodegradable time over the period of several months up to 2 years [11].

The relatively rapid decrease in the mechanical properties of PLA-based composites can be explained by two phenomena: (1) the initially rapid hydration at the polymer-reinforcing phase boundary breaking the bond between the matrix and reinforcement and the local stress concentration that inhibits the transfer of applied stress from the polymer to the reinforcement; (2) PLA swelling, which generates hydrostatic forces that can initiate cracks in the composite [12,13]. In addition, an amorphous phase that is formed at the surface of the polymer, quickly disintegrates (decomposes) and therefore easily absorbs water, causing faster polymer degradation [1]. The swelling process can be inhibited by various methods, e.g., mechanical blocking by hard powder particles, fiber reinforcement, plasma treatment and by increasing the adhesion of the fibers to the matrix by wetting their surface with a suitable chemical [14].

Currently, various methods of PLA modification are used. Mixing PLA with additives is usually a practical way to improve the properties and cost reduction of this polymer [10]. In the case of powder particles used in medical applications, the most frequently added to the PLA basis are: silver, magnesium, iron and steel, silicon, boron nitrile, hydroxyapatite and more both in the micro and nano-scale [15,16,17,18,19,20]. These additives are commonly used for different medical purposes, namely: antibacterial, degradation, magnetic or conductive properties, among others. Such parameters of powders as morphology, volume fraction, reactivity, surface area, melting temperature, and oxygen content have a strong impact on the overall metal-polymer composite properties. With regard to pure PLA, Mg and Fe powder micro-particles improve its mechanical properties, in particular the strength and the modulus [15,17].

It is well known that Mg is a very reactive metal and rapidly degrades in the body fluid environment, accompanied by hydrogen evolution. Pure Fe can be also treated as a degradable metal, causing no inflammation reactions, no local toxicity, and slowly corroding in the human body environment. However, a degradation rate of pure Fe for resorbable bone implants is relatively slow [17].

Metallic particles added to the PLA matrix change polymer morphology, mechanical, physicochemical, and thermal properties after storage in solutions due to complex degradation processes [21,22,23]. In the presence of water, ester groups are degraded, and as a result molecular weight is decreased and low molecular weight soluble oligomers and monomers are released [24]. Iniguez F. et al [23] tested the influence of water-ethanol solutions on crystallization and hydrolytic degradation of PLA. The Mg additive in the PLA-based composite can form an alkaline environment which further accelerates PLA degradation. Moreover, the Mg provide also an additional diffusion pathway for water molecules to further promote the PLA degradation [15,16].

Therefore, the mechanical properties, thermal stability, and degradability of PLA with the addition of two metal powders: 5% Mg and 5% Fe (wt.%) incubated in a 0.9% saline solution at 37 °C up to 180 days were studied and analyzed. In addition, the experiment was extended to PLA with polyethylene PE (HD polyethylene, 5% by mass) and pure PLA, as reference samples, to bring insights in the in-vitro degradation of the PLA composites, to shaping novel biodegradable materials for medical applications. Using rapidly reacting magnesium or slowly dissolving iron, it may be possible tailoring the degradation rate of metal-reinforced PLA-based composites.

## 2. Materials and Methods

### 2.1. Samples Preparation

**Figure 1:**
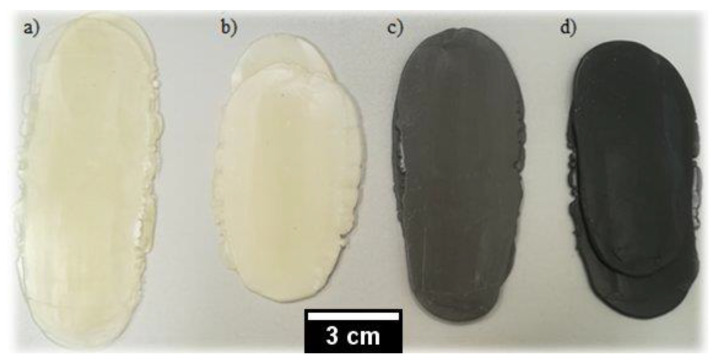
Prepared samples: (a) 100% PLA; (b) PLA-5% PE; (c) PLA-5% Mg; (d) PLA-5% Fe.

Three different compositions based on PLA were prepared with the addition of 5% Mg and 5% Fe in the form of powder particles with an average particle size about 45 μm (Johnson Matthey, London, UK) and 5% (wt.%) of highdensity polyethylene (PE-HD) in the form of granules with an average size of 3.5 mm (Borealis AG, Vienna, Austria), as a matrix reinforcement or blend. In order to compare the properties of the obtained composites, the test results were compared with pure polylactide (100% PLA, Nature Works, Minnetonka Blvd, MN, USA) obtained under the same process conditions described later. Mixtures of all composites were prepared by introducing modifiers into the PLA matrix using an EHP 25ELine laboratory extruder (Zamak Mercator, Skawina, Poland). The main parameters of the extrusion process are: the temperature of charge zone was 50 °C, the temperature of supply zone 170 °C, the temperature of compression zone 180 °C, the temperature of dispensing zone 180 °C, the extruder head temperature 175 °C, the extrusion speed was 5% of the maximum machine speed. After extrusion, the material strips, with approximately 100 mm long, 25 mm wide, and 4 mm thick, were further compressed (under the pressure of about 10 MPa) using a hydraulic press to get an equal thickness of 2.5 mm of flat samples, as shown in Figure 1.

Then, the degradability study of obtained materials (pure PLA and PLA composites) was conducted. Prepared composites were cut from the middle part into pieces with dimensions of 10 × 10 × 2.5 mm^3^ and three of them were incubated in the 0.9% saline solution with pH 6.37 ± 0.01. Plastic containers with samples were placed in an incubator with internal temperature of 37 ± 0.5 °C for a period of 180 days. After a certain period of time, weight loss of the specimens were tested as well as the pH and electrical conductivity of the saline solution.

### 2.2. Weight Loss Test

The degradation process of the prepared specimens was followed by determining the weight loss of the composite materials. Samples were washed with distilled water and gently air dried. The percentage of weight loss was determined after drying the samples by comparing dry weight (*w_d_*) with the initial weight (*w*_0_) according to Equation (1):

(1) $$weight\;loss\;\left(\%\right)=\;\frac{w_{0}-w_{d}}{w_{0}}\times 100\%$$

A balance (Mettler Toledo, Columbus, OH, USA) with a sensitivity of 0.01 mg was used.

### 2.3. Differential Scanning Calorimetry and Degree of Crystallinity

Differential Scanning Calorimetry (DSC) studies were carried out using a DSC Discovery apparatus (TA Instruments, New Castle, DE, USA). Tested samples in amount of 5 mg were placed in aluminum hermetic pans. The measurements were conducted in three cycles (heat–cool–heat), in a temperature range from −30 to 300 °C with a heating and cooling rate 10 °C/min and 5 °C/min, respectively, in the presence of purge nitrogen, with a flow rate of 10 mL/min. Note that results described in this work were taken from the second heating curves (first heating and cooling were performed to reduce the thermal history of the tested samples). Three samples of each material were tested. Obtained DSC curves were used to analyze the glass transition temperature (*T_g_*), crystallization temperature (*T_c_*), cold crystallization enthalpy (∆*H_c_*), melting temperature (*T_m_*), and fusion enthalpy (∆*H_m_*). The degree of crystallinity (*X_c_*) for the samples was determined according to the following Equation (2):

(2) $$X_{c}=\;\frac{∆H_{m}-∆H_{c}}{∆H_{m}^{\;\;\;\;\;100}}\times 100\%,$$

where ∆*H_m_*^100^ = 93.7 J/g is the fusion enthalpy of 100% crystalline PLA [25,26].

### 2.4. Thermogravimetry and Activation Energy

Thermogravimetry (TG) tests were carried out using a Q500 thermogravimetric analyzer (TA Instruments, New Castle, DE, USA) in a temperature range from 30 °C to 500 °C at nitrogen atmosphere with heating rates (*k*) of 5, 10, and 20 K/min. Special attention was paid to temperature at the start of thermal decomposition (*T_DS_*) (taken as 5% of the weight loss of the sample) and the end of thermal decomposition (*T_DE_*) (taken as 95% weight loss of the sample), and temperature at which the rate of weight loss was the highest (*T_max_*).

The Kissinger method was used to determine the activation energy of thermal decomposition of the tested materials. This method is based on the dependence of the temperature value *T_max_* (corresponding to the maximum of the TG signal) on the heating rate (*k)* according the Equation (3):

(3) $$ln\left(\frac{k}{T_{max}^{2}}\right)=-\frac{E_{a}}{R}\cdot \frac{1}{T_{max}}$$

As the heating rate increases, the temperature of the maximum intensity of the TG signal also increases. The Kissinger method is based on presenting the obtained *T_max_* values in the configuration *ln*(*k*/*T^2^_max_*) ≈ 1/*T_max_*. The directional coefficient of the obtained straight line corresponds to the value *E_a_*/*R* (where *E_a_* is the activation energy and *R*, is the gas constant equal to 8.31 J/mol∙K).

### 2.5. Hardness, Tensile Tests and SEM Observations

**Figure 2:**
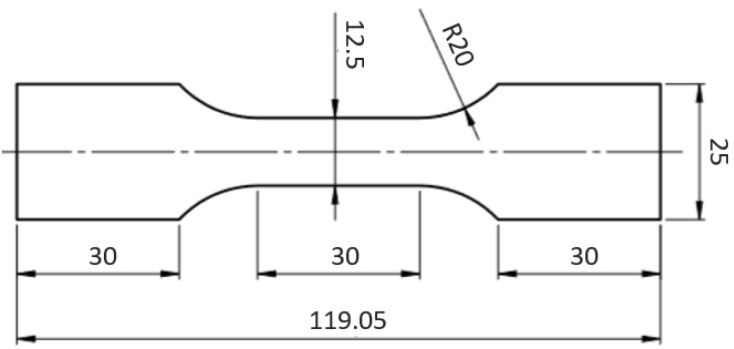
Dimensions of the sample used for tensile tests.

Hardness measurements of the composite materials were performed using a Shore durometer (Type D) according to ASTM D2240. Tensile tests were carried out with the MTS 858 Mini Bionix testing machines (extensometer measuring base of 25 mm) with a constant cross-head displacement rate of 10 mm/min, at room temperature. Five samples of each material were analyzed. The dimensions of samples for tensile testing are shown in Figure 2.

The tensile specimens were cut by means of a water-jet cutting machine (Kimla, Streamcut 978, Toronto, ON, Canada) toward longitudinal direction of the prepared composites. The thickness of the samples was 2.5 ± 0.15 mm. After tensile testing, fracture surfaces of the samples were observed by scanning electron microscope (SEM) S-3000N (Hitachi, Tokyo, Japan).

### 2.6. Physicochemical Properties of Contact Solution

During 180 days of the PLA samples incubation process at 37 °C the pH and electrical conductivity of 0.9% NaCl solution, were also measured. Initially, these parameters were tested every 7 days for a period of 30 days, then after 90, 120, 150 and 180 days, respectively. The pH and conductivity tests were carried out by means of suitable electrodes in conjunction with the multifunctional Seven-Multi ionic conductivity meter (Mettler Toledo; Greifensee, Switzerland). pH ratings were determined using a Clarytrode 120 electrode (Mettler Toledo, Columbus, OH, USA). Conductivity measurements were carried out using the InLab740 conductivity cell (Mettler Toledo, Columbus, OH, USA)) with an integrated probe for measuring the temperature of the tested solution. All tests were performed at the temperature of 21 °C.

## 3. Results and Discussion

**Figure 3:**
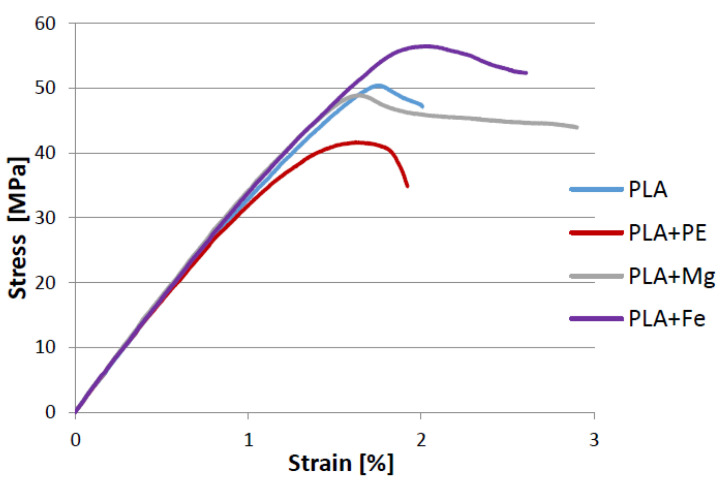
Typical curves of the tensile tested composite materials.

**Figure 4:**
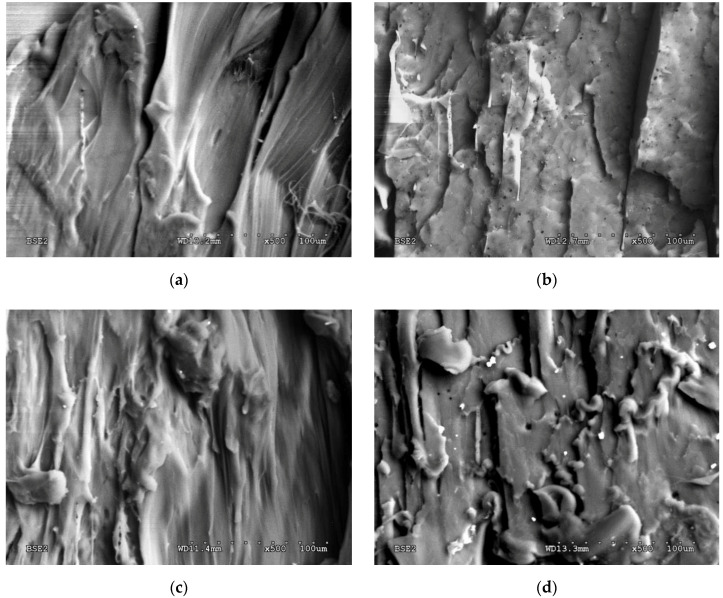

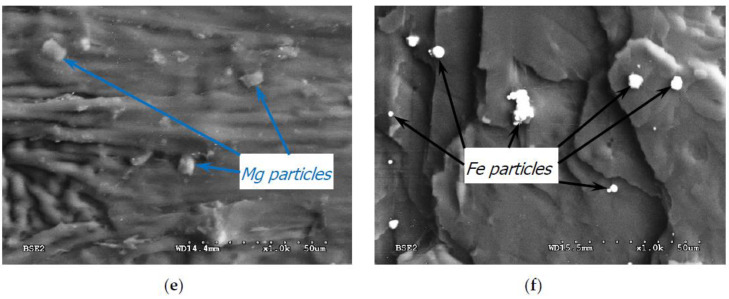
SEM images of the fracture surface of the broken composite samples obtained after tensile tests: (**a**) PLA, (**b**) PLA-5%PE, (**c**) PLA-5%Mg, (**d**) PLA-5%Fe (mag. × 500), (**e**) and (**f**) PLA-5%Mg and PLA-5%Fe powder particles, respectively (mag. ×1000).

Figure 3 presents an example of the tensile curves of each sample after extrusion and compression. Table 1 summarize the results of tensile strength together with Shore’s D hardness results. The fractured samples after tensile testing are shown in Figure 4.

Usage the high-density polyethylene as a blend with PLA as well as magnesium and iron powders as composite additives in comparison with the pure PLA shows changes in the mechanical properties of the tested materials. Note that all composite samples have slightly better ductility in comparison with the reference 100% PLA sample.

The highest Shore’s hardness, tensile strength (*σ*), yield strength *YS*_0.2_, and good total elongation at break (*ε*) were obtained for the PLA reinforced by iron (PLA-5%Fe), while the sample of PLA with magnesium (PLA-5%Mg) has the best ductility and comparable Young’s modulus (*E*) with the iron-based composite.

The introduction of PE-HD to PLA matrix caused, compared to the reference sample (pure PLA), decrease the tensile strength and elastic modulus of this material, while it slightly increased the elongation and hardness. Similar results were reported by Djellali S. et al. [26], however for a low density polyethylene (PE-LD) added to the PLA. The reason for this may be the flexible effect of the PE introduced to the PLA, resulting in a slight improvement in the plasticity [27]. However, the deterioration of the tensile strength of this material may be caused by a large difference in polarization between PLA and PE polymers, leading to their poor adhesion [28]. These suggestions were confirmed by SEM observations of the fracture surface of the samples discussed later in this article. Nevertheless, the microscopic observations of the all tested composite materials (not presented here) did not reveal any significant differences at the surface between the individual phases applied, and the obtained materials, especially PLA-5%PE, appeared to be macroscopically homogeneous.

It is worth noting that the Shore’a hardness of the composite materials is higher, compared to the pure PLA, while only the addition of PE caused the reduction of Young’s modulus by ~2.0%. The highest hardness was measured for the PLA-5%Fe sample, which increased in comparison to the pure PLA by about 7.5%.

The mechanical properties of composite polymers are strongly affected among others by dispersion of the reinforcing phase, matrix and reinforcing adhesion and filler aspect ratio. When the filler particles strongly impede the stretching of the polymer chains, it causes the decrease in elongation of the composites. On the other hand, it is assumed that improvement in tensile strength can be achieved after increasing load transfer between matrix and filler by increasing specific surface area of the powders and its homogeneity [26].

Figure 4 presents the fracture surface of all tested composite materials. Closer analysis of the fracture surface shows that a similar, fibrous nature of the fracture mode can be found for the pure PLA and PLA-5%Mg samples (see Figure 4a,c). Note, that the bonding between the PLA matrix and the reinforcement of the metallic powder particles seems to be strong and no areas of their clear detachment were visible. Nevertheless, singular pores were also observed.

The nature of both fractures can be described as fibrous and ductile. However, a different fracture surface was observed for the PLA-5%PE and PLA-5%Fe composites. In the case of the PLA-5%PE sample, clear facets, and typical detachment similar like for the brittle fracture of metallic materials (Figure 4b) are visible. The fracture of the PLA-5%Fe sample (Figure 4d) is flat with clear traces of delamination of individual layers, separated by the iron powder particles.

Spherical like Fe particles presented in Figure 4f, in comparison with more rectangular (flake-like) in shape of the Mg powder particles (see Figure 4e) were confirmed by SEM observations on the surface of both composite materials.

It is noteworthy that two metal powders with similar particle size mixed together with PLA polymer revealed a different nature of the destruction process during the static tensile testing. The tensile strength and Young modulus increase (see Table 1) can be related to the morphology, distribution and the degree of bonding of the metallic particles to the polymer matrix. The spherical Fe particles probably are stronger adhered to the polymer in comparison to the Mg particles that act as stress concentration points, thus facilitates PLA cracking and decreasing tensile properties of the PLA-5%Mg material. It is assumed that this state of affairs may also be affected by the thermal conductivity coefficient of both metals, which are different, and for a pure iron it is about 80 W/m·K, whereas for a magnesium it is two times higher and the values is 156 W/m·K. This parameter may significantly affects the thermal properties of both compositions, and thus the homogeneity of both materials after mixing with the screw feeder prior to extrusion, and consequently the mechanical properties of the obtained samples. This aspect, especially the total elongation increase in the PLA-Mg material, will be studied in detail in our future research work.

**Figure 5:**
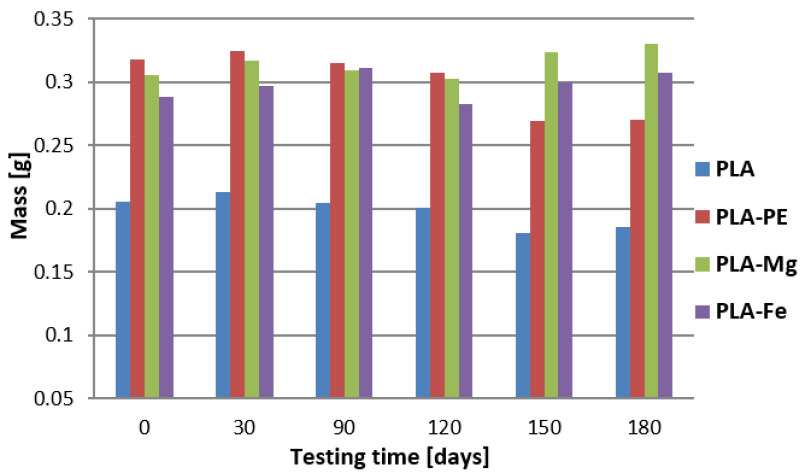
Degradation tests of the samples in the 0.9% saline solution.

Changes in mass of the tested samples immersed in the 0.9% saline solution, as a function of storage time, are shown in Figure 5. From these data it follows out that in the case of the pure PLA sample, the mass of the material initially increases up to 30 days, probably due to the rapid hydration of this polymer. After this time, a constant decrease in the mass of this sample is observed, which is in accordance with the literature data [29,30]. Similar results of the mass changes were measured for the sample with the PE addition, which indicates that this blend does not affect the PLA mass changing during storage it in the 0.9% saline solution.

**Figure 6:**
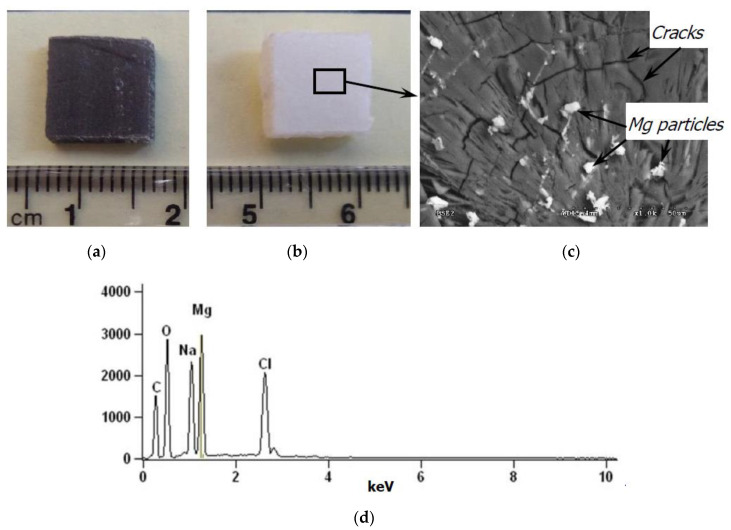
Elution of magnesium from the PLA-5%Mg composite (**a**) as-received, (**b**) after 120 days of testing, (**c**) SEM image of the PLA-5%Mg sample after 120 days of immersion (mag. ×1000), (**d**) SEM-EDS analysis of the Mg particles presented in Figure 6c (mag. ×1000).

A significantly different data of the weight loss over the testing time were measured for the PLA-5%Mg sample. In the initial period, up to 30 days, the mass of this material increases and then from 30 to 120 days decreases, and from 120 to 180 days increased again. Note that after 120 days of an immersion of this material, an elution of magnesium from the PLA matrix was observed, and the composite material itself changed color from initial dark grey to the color of milk, similar to the pure PLA sample (see Figure 6a,b). SEM-EDS observations in Figure 6c,d of the PLA-5%Mg after 120 days and drying revealed cracks of the PLA matrix and Mg particles attached with sodium chloride. With further extension of the degradation time in the 0.9% saline solution, the mass of this sample began to increase again, as a result of the penetration of the liquid by the capillary forces into the remaining pores after washing of the magnesium out. Note that PLA itself undergoes volumetric hydrolytic degradation, which constantly facilitates contact of the saline solution with the inner body of the material and can cause further elution of magnesium powder particles from the composite. This degradation caused some cracks (Figure 6c) similar to these reported by [31]. 

**Figure 7:**
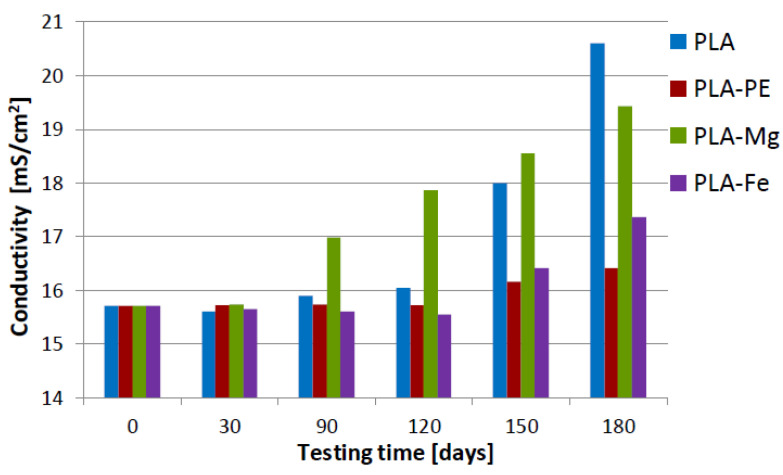
Results of the electrical conductivity of the saline solution after materials incubation up to 180 days.

In the case of the PLA-5%Fe composition, it is found that the degradability of this material has a sinusoidal course. In general, up to 90 days, the mass of this composite constantly increases. After these days up to 120 days the mass of the sample decreases, probably due to the effect of elution of the iron particles from the PLA matrix, to increase the mass again up to 180 days. This observation correlates well with the conductivity of 0.9% saline solution, in which this sample was incubated (as presented in Figure 7), where the values increased significantly from 16.05 ± 0.45 mS/cm after 120 days of testing time, to the value of 17.40 ± 0.10 mS/cm after 180 days of testing.

Relative mass changes (in %) of the tested samples were also calculated from the initial mass of the specimen and after 180 days of immersion samples in the contact solution. It was found that the highest relative mass loss calculated for the PLA-5%PE material (15.1%) and the lowest in the case of PLA-5%Fe (6.6%). This indicates that iron addition to the PLA matrix may be a good solution for improving PLA stability.

Thermal properties of tested pure PLA polymer and PLA composites before incubation and after incubation in 0.9% saline solution for 180 days are presented in Table 2. Analysis of obtained DSC spectra allowed for the determination of: the glass transition temperature (*T_g_*), crystallization temperature (*T_c_*), and melting temperature (*T_m_*), on the basis of which the crystallinity (*X_c_*) was calculated, both for first and second heating.

For all tested materials, changes in thermal properties were observed. *T_g_* and *T_c_* values of PLA and composites materials after first heating (I) are not significantly affected by the addition of Fe, Mg and PE probably because of their thermodynamic stability [17]. Note that the glass transition temperature for all tested samples were higher during first heating (I) comparing to second heating (II), i.e., *T_g_* = 57.4 °C for PLA (I, 0) and *T_g_* = 50.4 °C for PLA (II, 0), *T_g_* = 71.1 °C for PLA (I, 180) and *T_g_* = 43.0 °C for PLA (II, 180). For the tested materials it was observed that the value of *T_g_* was lower for samples incubated in the saline solution for 180 days in comparison to the control, which means that storage conditions affect the glass transition temperature. As the value of *T_g_* depends on different factors, such as molecular weight, intermolecular interaction, or chain flexibility [32], it can be concluded that hydrolytic degradation of PLA leads to decrease of molecular weight, which finally decreases the glass transition temperature. The highest differences were observed for the pure PLA and PLA-5%Mg samples. In the case of pure PLA kept in the saline solution, the glass transition temperature was 71.1 °C and for control *T_g_* = 57.4 °C, for PLA-5%Mg kept in 0.9% NaCl, the glass transition temperature was 70.5 °C, for control *T_g_* = 58.9 °C, respectively.

It is observed that, after 180 days of incubation of the specimens in the saline solution, the crystallization temperature decreased, indicating that, in the presence of the metal, powders shift the crystallization process to lower temperature. Observed decrease is probably due to hydrolysis of the polymer after storage in water environment, as was also reported by Ndazi et al. [33]. For control and after 180 days of incubation, it was appropriate: pure PLA *T_c_*(I) = 116.6 °C and *T_c_*(II) = 98.1 °C, PLA-5%PE *T_c_*(I) = 113.4 °C and *T_c_*(II) = 101.3°C, PLA-5%Mg *T_c_*(I) = 113.7 °C and *T_c_*(II) = 91.0 °C, PLA-5%Fe *T_c_*(I) = 105.8 °C, and *T_c_*(II) = 97.6 °C. On the other hand, an increase of the melting point temperatures of the crystalline phase was observed for all samples.

The melting temperatures were higher after incubation compared to control for all tested materials after first heating and lower in case of second heating due to increase of the degree of polymer crystallinity which occurs during the transformation of amorphous PLA into crystalline phases as the polymer was undergoing hydrolysis in water [33]. However, the difference between above temperatures were similar, i.e., for PLA *T_m_*(I, 0) = 148.8 °C and *T_m_*(I, 180) = 151.8 °C, for PLA-5%Fe *T_m_*(I, 0) = 146. 9 °C and *T_m_*(I, 180) = 151.7 °C.

Note that the degree of crystallinity after 180 days of incubation for all tested materials (pure PLA and PLA with the PE, Fe, and Mg additives) generally increase. Basing on enthalpy of cold crystallization and enthalpy of melting temperature, crystallinity of control samples and crystallinity of materials after incubation was calculated. As a result of the materials incubation, its crystallinity increases. Observed changes are significant, i.e., from the initial 13.35% to 30.65% for PLA, from 17.02% to 22.2% for PLA-5%PE, from 7.06% to 20.64% for PLA-5%Mg, and from 12.37% to 20.07% for PLA-5%Fe. The increase of crystallinity is usually associated with the formation of more ordered or bigger crystals. It was reported by other authors [34,35], PLA plasticizes and crystallizes in the presence of organic solvents, which cause polymer matrix swelling, increasing the chain mobility and solvent induced crystallization (SIC). Similar mechanism may occur in this work, where the water diffuse into material, caused swelling, plasticization, and finally, crystallization [36]. According to Wu et al. [35] in the first diffusion stage, the solvent molecules interact with random PLA coil chains, increasing the motion of the PLA segments, resulting either dissolution of the polymer or nucleation, leading to the rearrangement of PLA chains into crystal lattices.

Note that increase in the crystallinity results from the degradation mechanism of PLA. As it is known, the amorphous phase, which forms in the outer layer of the polymer, is particularly susceptible to water absorption and rapidly degrades. Hence, in the samples after certain degradation period, an increase in the crystallinity is observed, as a result in a decrease in the amount of the amorphous phase.

From TG curves, temperatures of thermal decomposition for 5% mass material loss (*T*_5%_) and for 95% mass material loss (*T*_95%_) were identified. Additionally, tests were performed at three different heating rates, namely: 5, 10, 20°C/min, for calculation of an activation energy of thermal decomposition (*E_a_*). Analysis of thermal decomposition shown that the temperature of thermal decomposition for 5% mass material loss (*T*_5%_) and for 95% mass material loss (*T*_95%_) was lower after 180 days of incubation for all tested materials except the PLA-5%PE sample. This indicates decomposition of the PLA after mixing with powders resulted in rupture polymer chains, that cannot be trapped by the metals particles, decreasing the thermal stability. It is expected, on the basis of the literature data [26,28] that metal powder nanoparticles can effectively trapped free radicals and thus increase the thermal stability of the composites. The decrease in *T*_5%_ and *T*_95%_ temperatures in the samples after the degradation period indicates a mechanism of chain breakage and the formation of shorter and lighter fractions, which are characterized by a lower temperature of onset and end of decomposition [30,37,38].

The activation energies of the control materials were lower in comparison to the samples tested after incubation in 0.9% saline solution. The highest differences were observed for following materials: (a) pure PLA: 73.3 kJ/mol (control) and 131.4 kJ/mol (after incubation), (b) PLA-5%PE: 109.6 kJ/mol (control) and 176.1 kJ/mol (after incubation), (c) PLA-5%Mg: 80.5 kJ/mol (control) and 139.8 kJ/mol (after incubation), (d) PLA-5%Fe: 88.1 kJ/mol (control) and 171.4 kJ/mol (after incubation). After incubation, observed activation energy increased, which means that more energy is required to initiate thermal decomposition of the tested materials. This phenomena was observed due to the increase of crystallinity of the tested materials after 180 days incubation process. In accordance with work of Taranie E. et al. [39] the increase of the activation energy is associated with the decrease of the mobility of polymer matrix and the lowering of the chemical reactivity of the corresponding chains.

**Figure 8:**
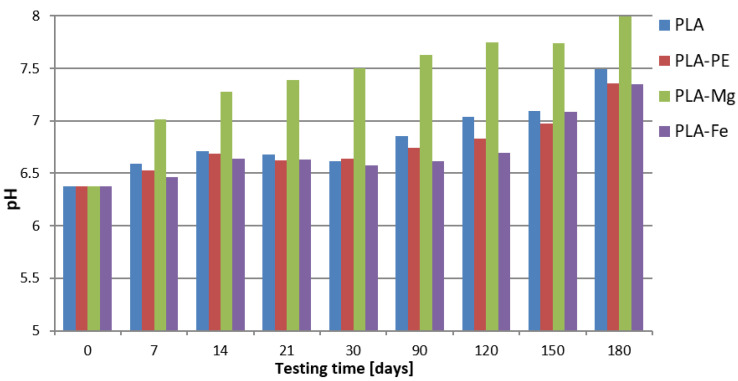
Results of the pH of the saline solution after materials incubation up to 180 days.

The results of pH data of the 0.9% saline solution as a function of the testing time are shown in Figure 8. In general, data presented in the graph revealed that the pH of the all samples increased, passing from a slightly acidic to a neutral pH or, as the data show after 180 days of testing time, clearly alkaline reaction.

Initially, the saline solution having reaction with PLA material release the free hydrogen ions in the process of electrolytic dissociation, and as a result of this reaction from the solution with the sample, oxygen ions evolved which, when combined with hydrogen ions, formed the hydroxyl groups, and the tested solutions changed the pH to alkaline. It is noteworthy to mention that the composite with the magnesium addition (PLA-5%Mg), after 7 days of testing time, has neutral pH of 7.01 ± 0.02 and after 180 days the pH of the solution is the highest in comparison to the all tested samples, and it is equal to 7.99 ± 0 01. This is due to the strong reactivity of this metal in the fluid environment.

As mentioned before, the correlation between the degradation process of the composite samples in the saline solution (the mass loss) and the electrical conductivity of the solution can be found. Each aqueous solution enriched in electric current carriers, namely ions, coming from the dissolved material, will have better electrical conductivity and therefore a higher value of Siemens parameter (S). In this context, two materials show a definite tendency to dissolve in the saline solution, pure PLA and PLA-5%Mg (Figure 7).

In the case of the composite with magnesium, there is about 8% increase in the electrical conductivity of the electrolyte, from 15.74 ± 0.24 (after 30 days) to 16.98 ± 0.12 mS/cm (after 90 days), so that after 180 days, the value of this parameter reached 19.42 ± 0.04 mS/cm.

Similar, not less interesting phenomenon can be observed in the case of pure PLA. The electrical conductivity of the contact solution oscillates slightly at the range of 15.7 ÷ 16.0 mS/cm up to 120 days, then suddenly increase after 150 and 180 days to the highest average value of 20.6 ± 0.05 mS/cm. This phenomenon can be explain by an abrupt change in the degree of crystallinity of the surface layer of the PLA, which as it is known the newly formed amorphous layer, has a distinct ability to a moisture absorption [3,40]. Due to this, the structure of the layer undergoes faster hydrolytic degradation, which leads to a decrease in the sample cross-section and, as a result, deteriorates its mechanical properties.

## 4. Conclusions

This paper presents the results of testing the composite materials based on polylactide with the addition of 5% PE, Mg and Fe (wt.%) obtained by an extrusion and subsequent pressing. The main purpose of this work was to find out whether, by using a metallic powder as a composite reinforcement, the mechanical properties of pure PLA could be improved and the degradation time can be control. This effect is very desirable for such materials used in medicine as well as in other industry applications.

In the course of the study, it was found that, compared to the pure PLA, an improvement in tensile strength (both σ and *YS*_0.2_) was obtained with the iron powder addition, while the magnesium powder slightly improved the ductility of the composite material (from 2.0% to 2.5%), at the expense of a minor reduction in the strength.

Studies on the degradation of these composites in the 0.9% saline solution over a period of 180 days revealed changes in the pH of the solution from acidic to alkaline, in all tested samples, after 150 days of conditioning at temperature 37 °C.

Similar mass changes were observed for the pure PLA and PLA-5%PE samples. The most varied mass loss was observed in the case of the PLA-5%Mg, where initially the sample mass increased (first 30 days) then decreased, and unexpectedly after 120 days the mass increased again and the sample changed the color, as a result of washing out the magnesium from the PLA matrix. This also caused the pH changes of the saline solution and an increase of the electrical conductivity. Similarly, a high increase in the electrical conductivity of the saline solution was found after 150 days for the pure PLA. In the context of degradation phenomenon of the tested materials, it turns out that after 180 days of immersing in the 0.9% saline solution the most stable is the PLA-5%Fe, which has also the highest tensile strength and hardness, whereas Mg addition caused the PLA composite has the highest reactivity and therefore short time of decomposition. 

## Figures and Tables

**Table 1:** Summarized results of tensile tests and hardness of the tested composite samples.

Sample	*σ* [MPa]	*ε* [%]	*YS*_0.2_ [MPa]	*E* [MPa]	*HS* [°Sh] (CV) *
100%PLA	50.1 ± 1.55	2.00 ± 0.29	49.2 ± 0.29	3466 ± 84.4	66.8 ± 0.55 (0.82)
PLA-5%PE	42.7 ± 1.25	2.05 ± 0.10	40.8 ± 1.64	3389 ± 121.1	68.4 ± 1.40 (2.02)
PLA-5%Mg	49.1 ± 0.35	2.55 ± 0.76	47.6 ± 1.04	3573 ± 51.8	70.1 ± 1.35 (1.92)
PLA-5%Fe	52.8 ± 2.75	2.15 ± 0.46	50.6 ± 2.39	3528 ± 76.2	71.7 ± 2.20 (3.04)

* CV—Coefficient of variation.

**Table 2:** Summarized results of DSC analysis: glass transition temperature (*T_g_*), crystallization temperature (*T_c_*), melting temperature (*T_m_*), and crystallinity (*X_c_*) obtained from DSC curves for first (I) and second (II) heating, and TG analysis: thermal decomposition temperatures for 5%_mas_. Material loss (*T*_5%_) and for 95%_mas._ material loss (*T*_95%_), and activation energy of thermal decomposition (*E_a_*). Results are presented for control samples (0) and for samples kept in 0.9% NaCl solution for 180 days (180).

Sample	*DSC_heating_*	*T_g_* (°C)	*T_c_* (°C)	*T_m_* (°C)	*X_c_* (%)	*T*_5%_ (°C)	*T*_95%_ (°C)	*E_a_* (kJ/mol)
PLA (0)	I	57.4	116.6	148.8	13.35	306.5	353.7	73.3
PLA (0)	II	50.4	119.6	146.4	0.46	-	-	-
PLA (180)	I	71.1	98.1	151.8	30.15	286.1	347.8	131.4
PLA (180)	II	43.0	107.7	143.9	1.48	-	-	-
PLA-PE (0)	I	56.5	113.4	148.1	17.02	303.4	382.9	109.6
PLA-PE (0)	II	55.8	117.0	148.2	7.5	-	-	-
PLA-PE (180)	I	57.6	101.3	153.1	22.2	290.1	363.9	176.1
PLA-PE (180)	II	45.7	100.4	146.7	9.13	-	-	-
PLA-Mg (0)	I	58.9	113.7	148.3	7.06	269.3	300.9	80.5
PLA-Mg (0)	II	46.3	106.4	142.7	3.8	-	-	-
PLA-Mg (180)	I	70.5	91.0	147.7	20.64	240.5	279.2	139.8
PLA-Mg (180)	II	16.4	83.1	143.7	1.52	-	-	-
PLA-Fe (0)	I	57.2	112.9	146.9	12.37	285.7	309.9	88.1
PLA-Fe (0)	II	48.7	116.4	146.4	0.24	-	-	-
PLA-Fe (180)	I	53.3	97.6	151.7	20.07	272.8	310.3	171.4
PLA-Fe (180)	II	37.9	104.5	139.0	2.91	-	-	-

## References

[1] Jalbrzykowski M. (2019). Selected Issues of the Injection Process of Biopolymers for Technical Applications, on the Example of Polylactide Acid.

[2] Liang H., Hao Y.P., Liu S.R., Zhang H.L., Li Y.S., Dong L.S., Zhang H.X. (2013). Thermal, rheological, and mechanical properties of polylactide/poly (diethylene glycol adipate). Polym. Bull..

[3] Piemonte V., Gironi F. (2013). Kinetics of Hydrolytic Degradation of PLA. J. Environ. Polym. Degrad..

[4] Luckachan G.E., Pillai C.K.S. (2011). Biodegradable polymers-a review on recent trends and emerging perspectives. J. Polym. Environ..

[5] Malinowski R., Rytlewski P., Żenkiewicz M. (2011). Effects of electron radiation on properties of PLA. Arch. Mater. Sci. Eng..

[6] Norazlina H., Kamal Y. (2015). Graphene modifications in polylactic acid nanocomposites: A review. Polym. Bull.

[7] Żenkiewicz M., Rytlewski P., Malinowski R. (2010). Compositional, physical and chemical modification of polylactide. J. Achiev. Mater. Manuf. Eng..

[8] Liber-Kneć A., Łagan S. (2014). Application of the measurement of the contact angle and the surface free energy to characterize the surfaces of polymers used in medicine. Polym. Med..

[9] Łysik D., Mystkowska J., Markiewicz G., Deptula P., Bucki R. (2019). The influence of mucin-based artificial saliva on properties of polycaprolactone and polylactide. Polymers.

[10] Domb A.J., Kumar N. (2011). Biodegradable Polymers in Clinical Use and Clinical Development.

[11] Wootthikanokkhan J., Cheachun T., Sombatsompop N., Thumsorn S., Kaabbuathong N., Wongta N., Wong-On J., Isarankura Na Ayutthaya S., Kositchaiyong A. (2013). Crystallization and Thermomechanical Properties of PLA Composites: Effects of Additive Types and Heat Treatment. J. Appl. Polym. Sci..

[12] Renouf-Glauser A.C., Rose J., Farrar D.F., Cameron R.E. (2014). The effect of crystallinity on the deformation mechanism and bulk mechanical properties of PLLA. Biomaterials.

[13] Muhammad Sami H. (2012). Phosphate Glass Fibre Reinforced Composite for Bone Repair Applications: Investigation of Interfacial Integrity Improvements via Chemical Treatments. Ph.D. Thesis.

[14] Jia S., Yu D., Zhu Y., Wang Z., Chen L., Fu L. (2017). Morphology, crystallization and thermal behaviors of PLA-based composites: Wonderful effects of hybrid GO/PEG via dynamic impregnating. Polymers.

[15] Cifuentes S.C., Frutos E., Benavente R., Lorenzo V., González-Carrasco J.L. (2017). Assessment of mechanical behavior of PLA composites reinforced with Mg micro-particles through depth-sensing indentations analysis. J. Mech. Behav. Biomed. Mater.

[16] Cifuentes S.C., Gavilán R., Lieblich M., Benavente R., González-Carrasco J.L. (2016). In vitro degradation of biodegradable polylactic acid/magnesium composites: Relevance of Mg particle shape. Acta Biomater..

[17] Jiang D., Ning F. (2020). Fused Filament Fabrication of Biodegradable PLA/316L Composite Scaffolds: Effects of Metal Particle Content. Procedia Manuf..

[18] Swain S.K., Gotman I., Unger R., Kirkpatrick C.J., Gutmanas E.Y. (2016). Microstructure, mechanical characteristics and cell compatibility of β-tricalcium phosphate reinforced with biodegradable Fe–Mg metal phase. J. Mech. Behav. Biomed. Mater..

[19] Tan L.J., Zhu W., Zhou K. (2020). Recent Progress on Polymer Materials for Additive Manufacturing. Adv. Funct. Mater..

[20] Ferri J.M., Motoc D.L., Bou S.F., Balart R. (2019). Thermal expansivity and degradation properties of PLA/HA and PLA/TCP in vitro conditioned composites. J. Therm. Anal. Calorim..

[21] Tsuji H., Sumida K. (2001). Poly (L-lactide): V. Effects of storage in swelling solvents on physical properties and structure of poly (L-lactide). J. Appl. Polym. Sci..

[22] De Jong S., Arias E.R., Rijkers D., Van Nostrum C., Kettenes-Van den Bosch J., Hennink W. (2001). New insights into the hydrolytic degradation of poly (lactic acid): Participation of the alcohol terminus. Polymer.

[23] Iniguez-Franco F., Auras R., Burgess G., Holmes D., Fang X., Rubino M., Soto-Valdez H. (2016). Concurrent solvent induced crystallization and hydrolytic degradation of PLA by water-ethanol solutions. Polymer.

[24] Tsuji H., Auras R.A., Lim L.-T., Selke S.E.M., Tsuji H. (2010). Hydrolytic degradation. Poly (lactic Acid). Synthesis, Structures, Properties, Processing, and Applications.

[25] Jalbrzykowski M., Krucinska I., Dabrowski J. (2016). Tests of selected mechanical properties of PLA-PLA type composites. Compos Theory Pract..

[26] Djellali S., Haddaoui N., Sadoun T., Bergeret A., Grohens Y. (2013). Structural, morphological and mechanical characteristics of polyethylene, poly (lactic acid) and poly (ethylene-co-glycidyl methacrylate) blends. Iran Polym. J..

[27] Abay A.K., Gebeyehu M.B., Lin H.K., Lin P.C., Lee J.-Y., Wu C.-M., Murakami R.-I., Chiang T.-C. (2016). Preparation and characterization of poly (lactic acid)/recycled polypropylene blends with and without the coupling agent, n-(6-aminohexyl) aminomethyl triethoxysilane. J. Polym. Res..

[28] Nisar M., Bernd M., Filho L., Geshev J., Galland G.B. (2019). Polypropylene/carbon nanotube magnetic composites obtained using carbon nanotubes from sawdust. Polym. Adv. Technol..

[29] Zhu J., Uhl F.M., Morgan A.B., Wilkie C.A. (2001). Studies on the mechanism by which the formation of nanocomposites enhances thermal stability. Chem Mater..

[30] Visakh P.M., Yoshihiko A. (2015). Thermal Degradation of Polymer Blends, Composites and Nanocomposites. Engineering Materials.

[31] Zeng R., Qi W., Song W.-Y., He Q.-K., Cui H.-Z., Han E.-H. (2014). In vitro degradation of MAO/PLA coating on Mg-1.21Li-1.12Ca-1.0Y alloy. Front. Mater. Sci..

[32] Qin L., Qiu J., Liu M., Ding S., Shao L., Lü S., Zhang G., Zhao Y., Fu X. (2011). Mechanical and thermal properties of poly(lactic acid) composites with rice straw fiber modified by poly(butyl acrylate). Chem. Eng. J..

[33] Ndazi B.S., Karlsson S. (2011). Characterization of hydrolytic degradation of polylactic acid/rice hulls composites in water at different temperatures. Express Polym. Let..

[34] Gao J., Duan L., Yang G., Zhang Q., Yang M., Fu Q. (2012). Manipulating poly (lactic acid) surface morphology by solvent-induced crystallization. Appl. Surf. Sci..

[35] Wu N., Lang S., Zhang H., Ding M., Zhang J. (2014). Solvent-induced crystallization behaviors of PLLA ultrathin films investigated by RAIR spectroscopy and AFM measurements. J. Phys. Chem. B.

[36] Elsawya M.A., Kimc K.-H., Parkc J.-W., Deep A. (2017). Hydrolytic degradation of polylactic acid (PLA) and its composites. Renew. Sustain. Energy Rev..

[37] Al-Itry R., Lamnawar K., Maazouz A. (2012). Improvement of thermal stability, rheological and mechanical properties of PLA, PBAT and their blends by reactive extrusion with functionalized epoxy. Polym. Degrada. Stabil..

[38] Hamad K., Kaseem M., Yang H.W., Deri F., Ko Y.G. (2015). Properties and medical applications of polylactic acid. Express Polym. Lett..

[39] Taranie E., Papageorgiou G.Z., Bikiaris D.N., Chrissafis K. (2019). Kinetics of Crystallization and Thermal Degradation of an Isotactic Polypropylene Matrix Reinforced with Graphene/Glass-Fiber Filler. Molecules.

[40] Krzan A. (2013). Biodegradable Polymers and Plastics, “Innovative Value Chain Development for Sustainable Plastics in Central Europe”. PLASTiCE. https://keep.eu/projects/5559/.

